# RTS,S/AS01_E_ vaccine defaults in Ghana: a qualitative exploration of the perspectives of defaulters and frontline health service providers

**DOI:** 10.1186/s12936-023-04690-4

**Published:** 2023-09-06

**Authors:** Joshua Okyere, Vincent Bio Bediako, Josephine Akua Ackah, Emmanuella Acheampong, Bernard Afriyie Owusu, Wonder Agbemavi, Adanna Uloaku Nwameme, Edward Mberu Kamau, Emmanuel Asampong

**Affiliations:** 1 Department of Population and Health, University of Cape Coast, Cape Coast, Ghana; 2 School of Nursing and Midwifery, College of Health Sciences, Kwame Nkrumah University of Science and Technology, Kumasi, Ghana; 3 The Graduate Group in Demography, University of Pennsylvania, Philadelphia, USA; 4 Department of Population Health, London School of Hygiene and Tropical Medicine, London, UK; 5 School of Demography, Australian National University, Canberra, Australia; 6 School of Public Health, University of Ghana, Accra, Ghana; 7 UNICEF/UNDP/World Bank/WHO Special Programme for Research and Training in Tropical Diseases (TDR) at the World Health Organization (WHO), Geneva, Switzerland

**Keywords:** Malaria, Vaccination, RTS,S/AS01_E_ vaccine, Default, Qualitative research

## Abstract

**Background:**

While Ghana has a good track record in the Expanded Programme on Immunization, there are substantial challenges with regards to subsequent vaccinations, particularly after the first year of life of the child. Given that the last dose of the RTS, S/AS01_E_ vaccine against malaria is administered at 24 months, there is a high likelihood of default. Hence, it is imperative to understand the dynamics and reasons for the defaults to enable the development of effective implementation strategies. This study explored why caregivers default on the RTS, S/AS01_E_ vaccine from the perspective of health service providers and caregivers.

**Methods:**

This study employed an exploratory, descriptive approach. Using a purposive sampling technique, caregivers who defaulted and health service providers directly involved in the planning and delivery of the RTS, S/AS01_E_ vaccine at the district level were recruited. A total of five health service providers and 30 mothers (six per FGD) participated in this study. Data analysis was done using NVivo-12 following Collaizi’s thematic framework for qualitative analysis. The study relies on the Standards for Reporting Qualitative Research.

**Results:**

Reasons for defaulting included the overlap of timing of the last dose and the child starting school, disrespectful attitudes of some health service providers, concerns about adverse side effects and discomforts, travel out of the implementing district, the perception that the vaccines are too many, and lack of support from partners.

**Conclusion:**

To reduce the occurrence of defaulting on the RTS, S/AS01_E_ vaccine programme, stakeholders must reconsider the timing of the last dose of the vaccine. The schedule of the RTS, S/AS01_E_ vaccine should be aligned with the established EPI schedule of Ghana. This will significantly limit the potential of defaults, particularly for the last dose. Also, the findings from this study underscore a need to encourage male partner involvement in the RTS, S/AS01_E_ vaccine programme. Health promotion programmes could be implemented to raise caregivers’ awareness of potential adverse reactions and discomforts—this is necessary to prepare the caregiver for the vaccine process psychologically.

**Supplementary Information:**

The online version contains supplementary material available at 10.1186/s12936-023-04690-4.

## Background

Over the past decades, there has been significant progress in the global fight against malaria. This progress is evidenced in the 62% decline in malaria-related deaths between 2000 and 2015 [1]. In developing countries like Ghana, the implementation of malaria preventive policies such as the National Malaria Control Programme (NMCP) increased the accessibility to free long lasting insecticidal nets (LLINs), and that contributed substantially to the progress seen in the fight to eradicate malaria [2]. For instance, a related study from Ghana [3] has shown that household access to LLINs alone resulted in a 7.1% reduction in self-reported malaria.

Notwithstanding the progress made over the years, malaria remains a global public health concern, with an estimated 241 million cases recorded in 2020 [2]. Evidence suggests that in 2021, the WHO African region contributed to 96% of the global malaria mortality, with children under-five accounting for nearly 80% of these deaths [2]. Ghana contributes to three percent of the global burden of malaria and is considered a high-burden country [4]. The unacceptably high burden of malaria in Ghana and across the globe has reinforced initiatives to end malaria. One such preventive initiative is the introduction of the RTS, S/AS01_E_ vaccine [5] to target children aged below 5 years and among whom the burden of malaria-related deaths is high.

Available evidence indicates that the RTS, S/AS01_E_ vaccine is the first vaccine approved to combat malaria [6]. The vaccine offers partial protection against malaria and has been found to reduce malaria incidence by 39% among children aged 5–17 months after taking all four doses [5]. Recognizing the vaccine’s modest efficacy, the World Health Organization (WHO), on April 24, 2017, announced measures to make the RTS, S/AS01_E_ vaccine available to three sub-Saharan Africa (SSA) countries: Ghana, Kenya, and Malawi [7]. This signalled the start of a pilot implementation of the RTS, S/AS01_E_ vaccine in SSA.

Through the Ministry of Health and Ghana Health Service, Ghana launched its Malaria Vaccine Implementation Programme (MVIP) in some selected districts in the then Brong Ahafo Region (now Bono, Bono East and Ahafo Regions), Central, Volta, and Upper East Regions [6, 8]. The vaccine was piloted in April, 2017 and later introduced into the national childhood immunization programme in May 2019 [9]. Ghana’s Expanded Programme on Immunization (EPI), funded through a joint effort between the government and other international partners like the Global Alliance for Vaccines and Immunizations (GAVI), ensures free and timely provision of vaccines for preventable infectious diseases in the early years of life [12]. Children receive about 13 vaccines and they are scheduled in the first 18 months after birth. In the 18th month, the last dose for Measles-Rubella and Meningococcal A conjugate vaccine are administered. The introduction of the RTS,S vaccine into this EPI schedule enhanced uptake and coverage, to align with that of other vaccines. The MVIP stipulated the RTS, S/AS01_E_ vaccine to be administered in four doses: at 6, 7, 9 and 24 months of age [6, 10]. While Ghana has a good track record in the EPI [6], there are substantial challenges with continuity, particularly after the first year of life of the child. For instance, the uptake of multiple-dose vaccines like Measles-Rubella tend to wane with time. In Northern Ghana, Dalaba and colleagues [11] reported a vast decrease between the first (95.3%) and second dose (18.2%) of the Measles-Rubella vaccine. Given that the last dose of the RTS, S/AS01_E_ vaccine, is administered at 24 months, 6 months after the last of the EPI (dose 2 of the Measles and Rubella vaccine), a high likelihood of default is only imminent.

Vaccination default refers to a situation whereby children “miss scheduled vaccinations for any reason, including health facility problems such as cancelled sessions or vaccine stock outs” [12]. High default rates can threaten the scale-up of the MVIP. Hence, it is imperative to understand the dynamics and reasons for the defaults to develop effective implementation strategies. Existing RTS, S/AS01_E_ vaccine implementation-related studies in Ghana have generally investigated challenges in planning and implementation [6], caregivers' willingness to pay for vaccines [13] and predictors of the uptake [14, 15]. Regarding uptake, studies have reported education, vaccine acceptance, confidence in the vaccine, rumours, logistics, and follow-ups as significant predictors [14, 15]. While these studies have provided significant insights, they have not entirely captured the subjective experiences underlying RTS, S/AS01_E_ vaccination defaults. Furthermore, study findings have been geographically limited to the Northern (Upper East) and Middle belt (Bono) regions of Ghana [14, 15], with little evidence from implementing districts in the South.

Defaulting is a distinctive aspect of vaccine uptake. An evaluation of the malaria vaccine implementation programme in Ghana has shown the default rate for dose one to three has been below 10%; however, the challenge is with the uptake of last dose of the vaccine [9]. The vaccine’s efficacy is dependent on the complete uptake of all doses. Failure to receive the full dose of the vaccine has the tendency to lead to reduced immunity against malaria, decreased effectiveness of the vaccine, and heightened vulnerability to malaria. It is, therefore, crucial to explore the inherent reasons for default to guide future programs and efforts to enhance vaccine completeness. Drawing on evidence from implementing areas in Southern Ghana (Central Region), the study a qualitative approach through the subjective lens of primary caregivers and healthcare providers to unearth the reasons for RTS, S/AS01_E_ vaccine defaults. The findings from this study are essential as they can feed into decisions, programmes and strategies that will be adopted towards a national scale-up shortly.

## Methods

### Study design

This study employed an exploratory, descriptive approach. The decision to use this study design was premised on the fact that the RTS, S/AS01_E_ vaccine is a new addition to the malaria preventive measures.

### Setting

**Fig. 1:**
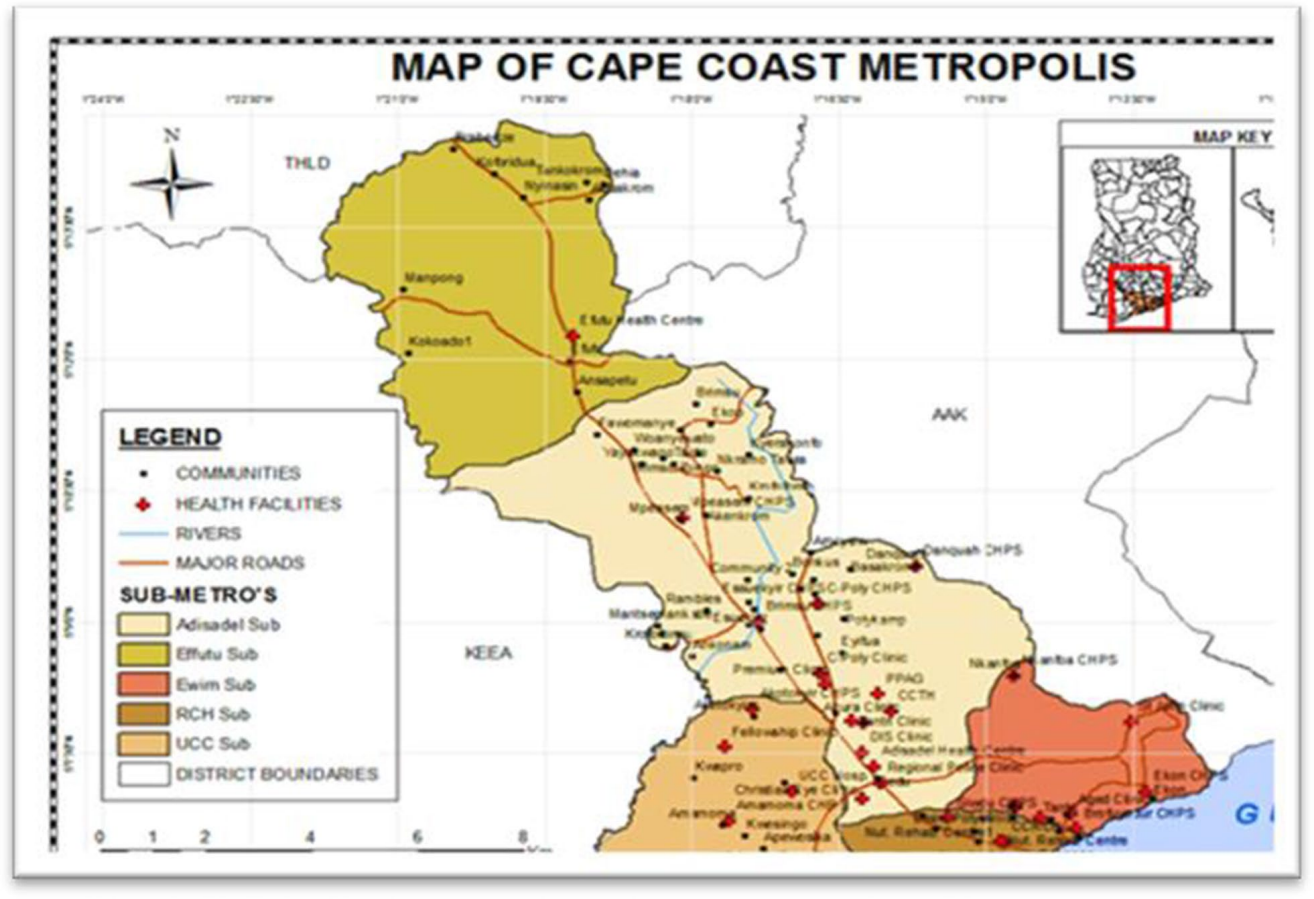
A map of Cape Coast Metropolis. Source: Cape Coast metropolis health directorate (2022), Cape Coast

The study was conducted within the Cape Coast Metropolis, located in the Central Region of Ghana. Cape Coast and five of its sub-metropolis are a part of the implementing districts for Ghana’s MVIP. These sub-metropolises are Ewin, Efutu, Adisadel, Cape Coast Central, and the University of Cape Coast. Below is a map of the metropolis showing all five sub-metros (see Fig. 1).

### Participant recruitment and sampling

Using a purposive sampling technique, health service providers directly involved in the planning and delivery of the RTS, S/AS01_E_ vaccine at the district level were recruited to participate as key informants for the study. The authors identified persons who defaulted on the RTS,S/AS01_E_ vaccination. This was done by requesting a list of mothers who had defaulted from the health service provider in charge of the district vaccination. The identified defaulters were then contacted by the team and invited to participate in focus group discussions (FGDs). A total of five health service providers and 30 mothers (six per FGD) participated in this study. After the fifth FGD, there was no new analytical information; thus, indicating the point of saturation.

### Data collection

Both key informant interviews (KIIs) and FGDs were conducted. The KIIs were conducted to understand the perspectives of health service providers directly involved in planning and delivering the RTS, S/AS01_E_ vaccine at the district level. Hence, for each district, one KII was conducted using a semi-structured interview guide. Similarly, for each district, one FGD was organized with mothers who had been identified as defaulters (i.e., those who missed scheduled vaccinations for any reason). The FGDs were conducted using an FGD guide. The semi-structured interview and FGD guides are attached as Additional files (see Additional file 1). In all, a total of five KIIs and five FGDs were conducted. Each FGD session was composed of six participants. The KIIs were conducted in the office of the health service provider, while the FGDs were conducted in a place that was agreed upon by all of the discussants. Prior to each interview, the rights of the participants were reiterated. The interviews with the health service providers were conducted in the English Language. However, the FGDs were conducted in the dominant local language (Fante). On average, each KII lasted 37 min, while the FGDs lasted about 60 min. All interviews were recorded using a tape recorder.

### Data analysis

Data analysis began with the transcription of all the audio data. A back-to-back translation was performed for the interviews conducted in the local language (i.e., Fante), while a verbatim transcription was done in English. The transcripts were imported into the QSR NVivo-12 to ease the data coding and extraction of the output. Following Collaizi’s thematic framework [16], the transcripts were read about three times to get familiarized with the data. Codes were inductively assigned. Patterns that emerged from the coding were categorized into themes. The authors had discussions to deliberate on the emerging issues and decide on the final categorization of the themes.

### Rigour and reflexivity

The reflexivity of this study is evidenced in the composition of the research team. The team was made up of population health scientists. None of the research team members had a relationship with the various health facilities. As a result, they did not influence the study participants or the general dynamics of the data collection. The study also attempted to maximize rigour by ensuring credibility, confirmability and transferability. Only verbatim quotations were used in reporting the perspectives of the study participants. This helped to ensure the study's credibility as it limited the likelihood of introducing interviewer biases. A detailed description of the research method guarantees the transferability of the study to similar socio-cultural contexts. For confirmability, there was an audit trail of the signed informed consent forms, transcripts, and audio data. The study relies on the Standards for Reporting Qualitative Research [17].

### Ethical approval

The Ghana Health Service granted ethical approval for this study (GHS-ERC: 006/09/22). Additional permission was also sought from health facilities. The methods align with the Helsinki and Belmont Declaration [18]. Both written and oral consent was obtained from all of the participants. The participants were provided with an information sheet detailing the study's objectives, duration, procedures, and potential discomforts and benefits; and their rights to confidentiality, privacy, and anonymity were emphasized.

## Results

### Participants’ characteristics

Table 1 summarizes the background characteristics of the frontline healthcare service providers. The frontline healthcare service providers were between 24 and 39 years, with a corresponding work experience of between 2 and 11 years. Regarding their educational qualifications, three of them had a certificate in community health nursing, while the remaining two key informants had a diploma.

**Table 1:** Background characteristics of key informants

ID	Age	Gender	Years of practice	Sub-districts	Qualification	Speciality
CHN 1	30	Female	7	Ewim	Certificate	Community health nurse
CHN 2	44	Female	11	Efutu	Certificate	Community health nurse
CHN 3	24	Female	2	Adisadel	Certificate	Community health nurse
CHN 4	39	Female	4	Cape Coast central	Diploma	Community health nurse
CHN 5	33	Female	4	University of Cape Coast	Diploma	Community health nurse

Table 2 presents a summary of the background characteristics caregivers. The caregivers were aged between 19 and 61 years. Most of the caregivers (n = 13) were married, while others were not married (n = 8), cohabiting (n = 7), or widowed (n = 2). The children cared for by these caregivers were aged between 1 and 3 years (see Table 2).

**Table 2:** Background characteristics of caregivers

FGDs #	ID	Age	Gender of caregiver	Age of child	Gender of child	Sub-districts	Marital status
1	R1	21 years	Female	1	Male	Ewim	Not married
	R2	28 years	Female	1	Male	Ewim	Married
	R3	33 years	Female	3	Female	Ewim	Not married
	R4	19 years	Female	2	Female	Ewim	Married
	R5	23 years	Female	2	Male	Ewim	Married
	R6	23 years	Female	1	Female	Ewim	Not married
2	R1	30 years	Female	2.5	Female	University of Cape Coast	Not married
	R2	33 years	Female	1	Male	University of Cape Coast	Cohabiting
	R3	27 years	Female	2.5	Male	University of Cape Coast	Cohabiting
	R4	29 years	Female	2.5	Female	University of Cape Coast	Cohabiting
	R5	31 years	Female	2	Male	University of Cape Coast	Married
	R6	24 years	Female	2	Female	University of Cape Coast	Married
3	R1	30 years	Female	3	Male	Adisadel	Married
	R2	36 years	Female	2	Female	Adisadel	Married
	R3	31 years	Female	3	Male	Adisadel	Married
	R4	29 years	Female	3	Male	Adisadel	Cohabiting
	R5	30 years	Female	2	Male	Adisadel	Married
	R6	27 years	Female	2	Female	Adisadel	Cohabiting
4	R1	30 years	Female	3	Female	Efutu	Widowed
	R2	27 years	Female	1	Male	Efutu	Not married
	R3	23 years	Female	2	Male	Efutu	Not married
	R4	27 years	Female	2	Male	Efutu	Not married
	R5	33 years	Female	2	Female	Efutu	Not married
	R6	61 years	Female	2	Male	Efutu	Widowed
5	R1	29 years	Female	2	Female	Cape Coast central	Cohabiting
	R2	33 years	Female	2	Male	Cape Coast central	Married
	R3	29 years	Female	1	Female	Cape Coast central	Married
	R4	30 years	Female	2	Male	Cape Coast central	Married
	R5	27 years	Female	1	Male	Cape Coast central	Cohabiting
	R6	30 years	Female	2	Male	Cape Coast central	Married

### Overview

The thematic analysis of the data from both the KIIs and FGDs unearthed the reasons why caregivers default on the malaria vaccine. The emerging themes were: the timing of the last dose and the child starting school, disrespectful attitudes of some health service providers, concerns about adverse side effects and discomforts, travelling out of the implementing district, the perception that the vaccines are too many, and lack of support from partners.

### Timing of last dose and child starting school

The timing of the last dose of the RTS, S/AS01_E_ vaccine, was one of the recurring factors reported by the participants concerning why they defaulted. From the perspective of the frontline service providers and caregivers, the gap between the third and final dose of the vaccine was too far apart. The participants asserted that they would have ordinarily completed their child welfare clinic schedules before the administration of the last the RTS, S/AS01E vaccine. Therefore, the large gap made it difficult for a caregiver to honour the vaccination schedules, hence, explaining why caregivers miss the final scheduled appointment for the RTS, S/AS01_E_ vaccine.

*“Sometimes, we forget that the child has to come for the vaccine. Especially during the last dose of the vaccine, you might forget about it because by then, we would have completed all the other vaccines given to children. So, we did not come to the child welfare clinic then. As such, it is very easy for us to forget that we must do something like this”* (R5, FGD 2, UCC).

Additionally, the participants stated that the 15 months gap between the third and final dose of the RTS, S/AS01_E_ vaccine was such that the child would have already been enrolled in school by the 24th month. This situation lessened the enthusiasm of caregivers to make their children available for the last dose of the RTS, S/AS01_E_ vaccine. One of the frontline service providers narrated:

*“For me, if you ask me why they default, I will say it is because of the timing of the shot, particularly the last one. We give the last shot at 24 months of the child. Many children would have started schooling then, and their mothers would have already resumed working. Thus, it becomes difficult for them to come for the last dose”* (CHN 2, KII, Efutu).

A participant from one of the FGDs also shared similar views about the timing of the last dose and the point that the child may have started schooling.

*“I think most mothers default for the last shot of the vaccine because, at that time, the child would have been enrolled in school. Moreover, I do not think parents will be enthused for their children to be vaccinated in school when they are not there to monitor how it is being done.”* (R1, FGD 2, UCC).

### Disrespectful attitudes of some health service providers

Multiple participants mentioned that caregivers default on the RTS, S/AS01_E_ vaccine due to some hostile attitudes exhibited by frontline healthcare service providers. The participants reported that some of the frontline healthcare service providers insulted and acted rudely towards them whenever they missed a scheduled appointment and later reported to the healthcare facility to make up for it. The caregivers, thus, interpreted this act of disrespect from the frontline service providers as embarrassing. Below are some narratives to buttress this point:

*“Some colleagues are rude to mothers who come here, especially when they miss their appointments. So, it becomes a disincentive for them to come because they are scared, they will be treated rudely.”* (CHN 3, KII, Adisadel).

*“I think that the attitudes of the health service providers are why some women default on the malaria vaccine uptake. When you sincerely forget and report there later than scheduled, some nurses can insult you and speak to you disrespectfully. To save themselves that embarrassment and disrespect, some who delay coming for the vaccine will not continue with it. So, they automatically become the defaulters.”* (R1, FGD 1, Ewim).

The disrespectful attitudes of the healthcare service providers were not only manifested when caregivers reported to the healthcare facility after a missed schedule. According to one of the caregivers, some healthcare service providers exhibited intolerance during their previous engagements while accessing child healthcare services.

*“In my view, the disrespectful nature of some health service providers is a turn-off for most mothers. Some nurses are not tolerant when we come for weighing [child welfare clinics]. The last thing and they would want to take advantage of that opportunity to disrespect you. Such behaviours do not encourage mothers to be compliant with the uptake of the malaria vaccine.”* (R3, FGD 1, Ewim).

### Concerns about adverse reactions and discomforts

While the participants agreed that the RTS, S/AS01_E_ vaccine was necessary to protect the child against malaria, they shared some concerns about the possible adverse reactions and discomforts associated with the vaccine's uptake. From the perspective of both the frontline healthcare service providers and the caregivers, the experience of adverse reactions such as abscess during any of the dosing phases of the vaccination programme served as a factor that influenced caregivers' decision to miss subsequent doses of the RTS, S/AS01_E_ vaccine.

*“Sometimes, when you administer the vaccine, the child will get an abscess. The site gets swollen, and it is excruciating for the child. Thus, some mothers default because they do not want their children to experience the pain. A certain woman came to complain that after receiving the third dose of the malaria vaccine, the child was paralysed, so she was not going to continue with the remaining dose of the vaccine.”* (CHN 3, KII, Adisadel).

One of the caregivers who had defaulted shared this sentiment:

*“I will talk about my own. I am a defaulter. I did not go for the third and last dose because of my child's reaction after the second dose. The child’s leg got swollen because of the vaccination, so the child could not walk. Because of that, I decided not to come for the remaining dose.”* (R3, FGD 2, UCC).

Some caregivers defaulted because of the negative experiences of other people concerning other vaccinations. Individuals whose parents or close social ties had experienced some adverse reactions due to a vaccine uptake were often reported to miss the RTS, S/AS01_E_ vaccine schedules.

This assertion is evidenced by a quote from one of the FGDs conducted:

“*I defaulted because of what happened to mother. My mother is physically disabled because the person who administered the vaccine wrongly did it when she was a child. In my case, I did not know that was my mother’s story. If I did, I would not have accepted it in the first place. Thus, when I learned about it, I decided not to come for the remaining doses.”* (R1, FGD 4, Efutu).

### Travelled out of the implementing district

The RTS, S/AS01_E_ vaccine programme in Ghana was implemented in some selected districts and sub-districts. This implied that not everyone was eligible to participate in the programme. The analysis revealed that some caregivers travelled out of the implementing districts where they had to receive some doses of the RTS, S/AS01_E_ vaccine. In such instances, those who travelled to districts that were not captured as implementation districts automatically defaulted because they could not get access to the vaccines.

*“Some women too may have defaulted because they travelled out of this place. You know, you cannot blame them [mothers] because the RTSS was a pilot, so it was not provided in all regions. So, women who did not complete their dose and travelled from this place defaulted.”* (CHN 2, KII, Efutu).

This assertion is also corroborated by the views of the caregivers who had defaulted:

*“Sometimes, you may be transferred from the community to another place or might even travel. When that happens, you will not be able to take the child to the clinic for vaccination. So, you end up defaulting.”* (R6, FGD 2, UCC).

*“Some mothers become defaulters because they might have travelled away from the district. Besides, the vaccine was only provided in some selected districts. Thus, if you travel with your vaccination card, you will still not find a place to get the shot.”* (R4, FGD 3, Adisadel).

### Perception that the vaccines are too many

Many participants expressed that caregivers defaulted because of the perception that too many vaccines were being administered to the children. Every child is expected to receive a series of vaccines for a host of diseases, including chickenpox, diphtheria, tetanus, and pertussis (DTaP), haemophilus influenza type b disease, measles, mumps, and rubella (MMR), polio, pneumococcal disease, hepatitis A, hepatitis B, and influenza. With the addition of the RTS, S/AS01E vaccine, caregivers perceive that children are overwhelmed and overburdened with vaccines. Thus, leading to missed RTS, S/AS01_E_ vaccine schedules.

One of the caregivers contended,

‘‘The vaccinations are too much for the children. Can’t they make the vaccine so that the child will take the malaria vaccine just once or twice? Because the four times that you have to come is emotionally damaging.”

(R4, FGD 4, Efutu).

Some frontline healthcare service providers reaffirmed this positionality of some caregivers:

*“The main challenge we face is compliance from mothers. Some mothers have the perception that the vaccinations are too many.”* (CHN 1, KII, Ewim).

*“There are also women who defaulted because they thought the vaccines were too much for the child. As I said, the child is expected to take the BCG, polio, yellow fever, penta, pneumococcal and the rotavirus vaccine. Then, they have to take four doses of RTSS. Some mothers see it to be synonymous with burdening the child. Thus, they default when they feel they have had enough.”* (CHN 2, KII, Efutu).

### Lack of support from partners and family

Within the Ghanaian socio-cultural system, the normative values ascribed by the society place much premium on men's decision-making power and authority as the head of the family or household. For that reason, issues relating to healthcare decisions often require that caregivers (primarily mothers) consult and gain their partners' support to seek healthcare, including meeting the RTS, S/AS01_E_ vaccine schedules. The participants, however, indicated that there were times when their partners were not supportive. Their partners did not provide them with the necessary financial resources to cater for the cost of their transportation to the child welfare clinic for the RTS, S/AS01_E_ vaccine.

*“Some of the women will tell you that their husbands refused to give them money, and so they did not have money for transportation, that is why they did not show up for their appointment date”* (CHN 3, KII, Adisadel).

Another perspective to this argument was that male partners refused to take over the responsibility of taking the child to the healthcare facility when mothers were constrained by their work demands to fulfil their obligations. Moreover, some of the caregivers who defaulted had no extra support from their families and friends.

*“Some mothers are so busy with work that they have no time to spare to take the child for vaccination. The men will not even sacrifice their time to send the child to the facility for the vaccination”* (R4, FGD 2, UCC).

*“Most times, they default because they are extremely busy with their work and other responsibilities so much that vaccination becomes a secondary thing for them. We do not have anyone to support us with the child’s health needs”* (R4, FGD 3, Adisadel).

## Discussion

The RTS, S/AS01_E_ vaccine has helped reduce malaria-related morbidity and mortality among children aged below 5 years in implementing countries in Africa. Based on reports from the previous phase 3 trial, the vaccine’s overall protection against all malaria episodes was higher if all four doses were taken [19]. Unfortunately, the vaccine uptake keeps reducing with an increasing number of doses. Evidence from a study in Ghana showed that the uptake reduced to about 58 percent at dose 4 [14]. This means that a substantial number of primary caregivers default over time. Having missed opportunities result in reduced protection and, subsequently, more malaria-related morbidities. The findings provided insights into the underlying reasons for the RTS, S/AS01_E_ vaccine defaults.

For most mothers, the timing of the fourth dose presents a major challenge. Since the last vaccine in the EPI (Measles and Rubella (MR) vaccine dose (2) is taken in the 18th month, uptake of the fourth dose of the RTS, S at 24 months is affected. Often, mothers are not obliged to attend child welfare clinics at health facilities after the MR dose 2. They may only do so to check the child’s weight and height or to get additional health information, which may not necessarily be a priority. Mothers may, therefore, forget to send their children to the facility without constant reminders from healthcare providers.

Furthermore, some mothers enrol their young wards in day care centres as part of their work resumption plan and there is also the need to prepare children for pre-kindergarten. The timing of their ward’s “schooling” makes it challenging to abide by the schedule of the fourth dose. Even with the potential solution of in-school vaccination, mothers were uncomfortable about the vaccine being given in their absence. A re-scheduling of the fourth dose is, therefore, paramount. In the last quarter of 2022, the WHO shared lessons from the pilot of the vaccine and revealed an update on Ghana’s preparedness to re-set the schedule of the fourth dose to coincide with the country’s schedule for dose 2 of the MR vaccine [20]. It is currently unclear whether this has materialized in all implementing health facilities. The Ghana Health Service can work with the School Health Education Programme (SHEP) coordinators to leverage the school as a medium to remediate the defaults on the fourth dose of the RTS, S/AS01_E_ vaccine. Additionally, catch up and defaulter tracing initiative can be implemented at the community level to track and administer the RTS, S/AS01_E_ vaccine to children who may have missed their last dose. Furthermore, health workers need to be trained on how to best sensitize caregivers on number of doses while keeping their interest to complete the schedule.

The negative attitudes of healthcare providers as a disincentive for maternal and child healthcare utilization has previously been documented. Across sub-Saharan Africa, this constitutes a primary reason for child immunisation programme setbacks and the RTS, S/AS01_E_ vaccine programme is not an exception [14, 21]. Insults, impatience, and intolerance are some commonly mentioned patient complaints which have led to several recommendations, including training healthcare providers to promote efficient public relations. While this may be essential, it is not holistic in achieving overall effectiveness. Staff shortage is a concern in most African countries. In Ghana, the ratio is 2.7 and 0.1 per 1000 people for nurses/midwives and physicians, respectively [22, 23]. As a result, burnout and poor service quality become a maladaptive coping mechanism. Undoubtedly, the patient’s perspective is only one side of an important story. In order to fully comprehend the context and institutional factors that aggravate attitudes, more research is needed to investigate them and provide solutions.

All four doses of the RTS, S/AS01_E_ vaccine, are administered intramuscularly. Consequently, it comes with pain, swelling around the injection site and even possible disabilities, mainly when wrong injection procedures are performed. Similar concerns have been raised for other injectable vaccines [24, 25] and so these are not specific to the RTS, S/AS01_E_ vaccine. Irrespective of this, it is distressing for mothers to see their children endure pain or become paralysed. It is exacerbated by the increased number of vaccines and thus this becomes a deterrent. Other conditions, such as febrile convulsions, cerebral malaria and meningitis, were found to be adverse side effects during the phase 3 trial [26]. However, as found in this study, mothers’ descriptions of the side effects are not symptoms of these conditions. While this may be potential evidence of reducing these adverse side effects, the findings lack generalizability and quantitative validity. The study emphasizes the need for more research on children with these adverse conditions in local contexts. Meanwhile, supplementary training for healthcare providers on administering intramuscular vaccines is indispensable. Additionally, a future version of the RTS, S/AS01E vaccine could consider an oral form to dispel concerns and ensure that mothers do not feel their children have to suffer the discomfort of injections. Also, alternative vaccine delivery mechanisms such as intranasal delivery methods should be considered and thoroughly researched to ease the discomforts of receiving injections.

The RTS, S/AS01_E_ vaccine is currently implemented in only some selected districts. Caregivers’ migration out of such districts to resettle in non-implementing districts constitutes one of the reasons for defaulting since the vaccine will no longer be available. Even in cases where they resettle in another implementing district, mothers may miss the appropriate timing or even forget. This confirms the findings by Grant et al*.* [6], Yeboah et al*.* [14], and Tabiri et al*.* [15], who reported that some children in Ghana missed out on some doses because they travelled out of their implementing district. In a systematic review by Bangura et al*.* [27], migration was an important factor for low vaccination rates in sub-Saharan Africa. Missed opportunities resulting from migration are a difficult challenge to address, and future attempts for national scale-ups can only be relied on to address the issue.

Family plays a vital role in children’s upbringing, growth, development and even survival [28]. The support of family members, especially husbands/partners, is vital, and mothers who participated in this study drew attention on their absence and lack of support as a reason for defaulting the last dose of the RTS, S/AS01_E_ vaccine. The reason for the low participation of family members is varied. In some instances, husbands/partners [29] and other relatives living in households may generally be aware of child immunization and its benefits but not necessarily the various vaccines and the time schedules. In other instances, institutional health factors serve as deterrents. One of the significant findings from a systematic review of the determinants of male involvement in maternal and child health services in sub-Saharan Africa was the lack of space to accommodate male partners [27]. There is also the concern of an under-prioritization of the broader family as an influential audience in child health policies, programmes and interventions [30]. The study suggests a move beyond just awareness and education on health benefits to the active involvement of families in these programmes so that mothers can get the support they need. Government and non-government support will be needed to ensure special programmes are implemented to encourage families to participate in child immunization programmes, particularly the RTS, S vaccine, and the provision of infrastructures and physical spaces at appropriate health facilities.

## Strengths and limitations

As a study that relied on a qualitative research approach, its strength lies in its deep insights concerning why RTS, S/AS01_E_ vaccination defaults exist. The team’s deliberations on the codes and categorization of the themes ensured that the analysis was not shaped only through the lens of a single researcher interpreted the data. Notwithstanding, given that the study was limited to only one implementing region, the findings might not necessarily reflect the situation in the other implementation regions and their corresponding districts.

## Conclusion

This study has identified several factors that explain RTS, S/AS01_E_ vaccine default among caregivers in Ghana. To reduce the occurrence of defaulting on the RTS, S/AS01_E_ vaccine programme, stakeholders must reconsider the timing of the vaccine's last dose to align with Ghana's established EPI schedule. This will significantly limit potential defaults, particularly for the last dose. Also, the findings from this study underscore a need to encourage family involvement (particularly husbands/partners) in the RTS, S/AS01_E_ vaccine programme. Health promotion programmes could be implemented to raise caregivers’ awareness of potential adverse reactions and discomforts–this is necessary to prepare the caregiver for the vaccine process psychologically. Another health promotion programme could include sending reminder messages to the caregivers close to the vaccination date. The evidence from this research is also helpful for general programmes and planning for other vaccines in the EPI schedule.

### Supplementary Information

**Additional file 1: **Data collection instruments.

## Data Availability

Data for this study is available from the authors upon reasonable request.

## Abbreviations

EPI: Expanded programme on immunization
FGD: Focused group discussion
KIIs: Key informant interviews
MVIP: Malaria vaccine implementation programme
WHO: World Health Organization
SSA: Sub-Saharan Africa
