# Supplementary material for: RTS,S/AS01E vaccine defaults in Ghana: a qualitative exploration of the perspectives of defaulters and frontline health service providers
Source: Malar J. 2023 Sep 6;22:260. doi: 10.1186/s12936-023-04690-4 (PMC10483715; doi:10.1186/s12936-023-04690-4)
Supplement: Supplementary file 1 — Additional file 1: Data collection instruments. [file 12936_2023_4690_MOESM1_ESM.docx]

| **IDI WITH NURSES** | |
| --- | --- |
| **ALTERNATIVE IMPLEMENTATION STRATEGIES FOR THE ADMINISTRATION OF THE FOURTH VACCINE DOSE OF THE RTS,S IN THE CAPE COAST METROPOLIS** | |
|  | **Opening statements**  **Please note that this is just an outline, do not read out all the questions**  Thank you for agreeing to take part in this research interview. My name is _______ and I am a member of the team working on this study. Today we will have a conversation about child welfare clinic and the malaria vaccine (RTS,S).  You can pause or stop the interview at any point or skip any questions should you wish to. If anything I ask isn’t clear, or you would like time to think about your answer, or you would like to raise something that you think is important, then please do tell me.  What we talk about will not be shared directly and I will take out any names or places so that when I describe what family members/friends have told me, they will not be able to know who I have interviewed. Also I will not share anything directly about you with your care team. However, if I am worried about your safety or anyone else’s safety then I will let your care team know and I will let you know that I am doing that.  Could I please just confirm that you have signed consent form and read the information sheet? And are you happy for the interview to be audio recorded today? Do you have any questions before we start?  Ok, I am turning on the recorder -  **(START RECORDING)** |
|  | **SECTION 1: Warm up**   1. Tell me about yourself.   Ask for:   - Age of mother - Marital status - Age of child(ren) - Years of practice - Place of residence - Highest educational attainment - Specialty  1. What are some health problems that affect children you see at [health facility]?  - Probe: most pressing health concern. - Ask about malaria.  1. Which are the serious problems? How so? Why do you say this? 2. Now we would like to talk a bit about malaria. How does malaria affect people in this community? 3. How do you prevent children who come to your facility from being affected by these health problems? |
| **Section 2:** **Vaccine services** | |
|  | 1. What can you tell us about vaccination services at this health facility? 2. What are some challenges to delivering vaccination services at this health facility?  - Probe for: defaulting mothers  1. What approaches are adopted for child vaccination in this facility?  - Probe for: home care, through child welfare clinics, etc. |
| **Section 3:** **RTS,S vaccination** | |
|  | 1. Now let’s talk a bit about the malaria vaccination. How would you describe the purpose of the RTS,S vaccinations? 2. What do you see as the vaccine’s main advantages for child health in your community? 3. What implementation strategies are currently adopted for administering RTS,S vaccine in this facility?  - Probe for: home care, through child welfare clinics, etc.  1. Which of the implementation strategy for administering RTS,S vaccine has been most effective? Which approach has been less effective? |
| **Section 6: RTS,S default** | |
|  | 1. Now let us talk about why you defaulted on the RTS,S vaccine. Why do some women default on the RTS,S vaccine?   Probe for:   - Concerns about side effects - Distance and cost of visiting healthcare facility - Inconvenience in approach of vaccine administering  1. What alternative implementation strategies can be employed to improve on the number of children that receive their recommended vaccinations on time? |
|  | **Closing statements**  If there is anything you would like to add about your experiences of care or anything we have missed out/not spoken about, you can always contact us afterwards. I’d like to thank you for taking the time to be interviewed today, we really appreciate it and your views will be a great help to us. |
|  | **(END RECORDING)** |

| **Interview ID#:** | _________________  District | ________________________________  Community | |
| --- | --- | --- | --- |
| **Interview Date:** | ______ | ______ | ______ |
|  | D D | M M | Y Y |
| **Interviewer:** |  | **Transcriber:** |  |

| **FGD WITH NURSES** | |
| --- | --- |
| **ALTERNATIVE IMPLEMENTATION STRATEGIES FOR THE ADMINISTRATION OF THE FOURTH VACCINE DOSE OF THE RTS,S IN THE CAPE COAST METROPOLIS** | |
|  | **Opening statements**  **Please note that this is just an outline, do not read out all the questions**  Thank you for agreeing to take part in this research interview. My name is _______ and I am a member of the team working on this study. Today we will have a conversation about child welfare clinic and the malaria vaccine (RTS,S).  You can pause or stop the interview at any point or skip any questions should you wish to. If anything I ask isn’t clear, or you would like time to think about your answer, or you would like to raise something that you think is important, then please do tell me.  What we talk about will not be shared directly and I will take out any names or places so that when I describe what family members/friends have told me, they will not be able to know who I have interviewed. Also I will not share anything directly about you with your care team. However, if I am worried about your safety or anyone else’s safety then I will let your care team know and I will let you know that I am doing that.  Could I please just confirm that you have signed consent form and read the information sheet? And are you happy for the interview to be audio recorded today? Do you have any questions before we start?  Ok, I am turning on the recorder -  **(START RECORDING)** |
|  | **SECTION 1: Warm up**   1. Tell me about yourself.   Ask for:   - Age of mother - Marital status - Age of child(ren) - Years of practice - Place of residence - Highest educational attainment - Specialty  1. What are some health problems that affect children you see at [health facility]?  - Probe: most pressing health concern. - Ask about malaria.  1. Which are the serious problems? How so? Why do you say this? 2. Now we would like to talk a bit about malaria. How does malaria affect people in this community? 3. How do you prevent children who come to your facility from being affected by these health problems? |
| **Section 2:** **Vaccine services** | |
|  | 1. What can you tell us about vaccination services at this health facility? 2. What are some challenges to delivering vaccination services at this health facility?  - Probe for: defaulting mothers  1. What approaches are adopted for child vaccination in this facility?  - Probe for: home care, through child welfare clinics, etc. |
| **Section 3:** **RTS,S vaccination** | |
|  | 1. Now let’s talk a bit about the malaria vaccination. How would you describe the purpose of the RTS,S vaccinations? 2. What do you see as the vaccine’s main advantages for child health in your community? 3. What implementation strategies are currently adopted for administering RTS,S vaccine in this facility?  - Probe for: home care, through child welfare clinics, etc.  1. Which of the implementation strategy for administering RTS,S vaccine has been most effective? Which approach has been less effective? |
| **Section 6: RTS,S default** | |
|  | 1. Now let us talk about why you defaulted on the RTS,S vaccine. Why do some women default on the RTS,S vaccine?   Probe for:   - Concerns about side effects - Distance and cost of visiting healthcare facility - Inconvenience in approach of vaccine administering  1. What alternative implementation strategies can be employed to improve on the number of children that receive their recommended vaccinations on time? |
|  | **Closing statements**  If there is anything you would like to add about your experiences of care or anything we have missed out/not spoken about, you can always contact us afterwards. I’d like to thank you for taking the time to be interviewed today, we really appreciate it and your views will be a great help to us. |
|  | **(END RECORDING)** |

| **Interview ID#:** | _________________  District | ________________________________  Community | |
| --- | --- | --- | --- |
| **Interview Date:** | ______ | ______ | ______ |
|  | D D | M M | Y Y |
| **Interviewer:** |  | **Transcriber:** |  |
